# Acupoint transcutaneous electrical nerve stimulation in hospitalized COPD patients with severe dyspnoea: study protocol for a randomized controlled trial

**DOI:** 10.1186/s13063-019-3757-x

**Published:** 2019-12-11

**Authors:** Carles Fernández-Jané, Jordi Vilaró

**Affiliations:** 1 School of Health Science Blanquerna, Ramon Llull University, Padilla 326-332, 08025 Barcelona, Spain; 2 Global Research on Wellbeing (GRoW) Research Group, Ramon Llull University, Padilla 326-332, 08025 Barcelona, Spain

**Keywords:** AcuTENS, Acupuncture, COPD, AECOPD, Dyspnoea, Randomized control trial, Protocol

## Abstract

**Background:**

Acute exacerbation of chronic obstructive pulmonary disease (AECOPD) is a major cause of hospital admissions and dyspnoea is its main symptom. Some studies have concluded that a new modality of acupuncture, called acupoint transcutaneous electrical nerve stimulation (acuTENS), could reduce dyspnoea in patients with COPD by increasing β-endorphin levels. However, those trials have been conducted mainly on patients in stable condition. This study aims to determine whether the administration of acuTENS can reduce dyspnoea in patients hospitalized for AECOPD.

**Methods:**

A multicentre randomized control trial with blinding of participants and assessors will be conducted. A sample of 60 patients will be randomly assigned to receive 45 min of either real acuTENS or sham acuTENS treatment once a day for five consecutive days. The trial will be conducted at the “Hospital del Mar” in Barcelona (Spain) and the “Hospital Sant Joan de Déu de Manresa” in Manresa (Spain). The Borg scale at baseline and days 1 to 5 will be the primary outcome. Secondary outcomes will be the duration of the hospitalization, quantity of drugs administrated, expiratory peak flow adverse effects and mortality and readmissions at 3 months.

**Discussion:**

AcuTENS is a non-pharmacological, non-invasive and inexpensive intervention. This trial will help to elucidate the potential role of acuTENS in the treatment of AECOPD.

**Trial registration:**

ClinicalTrials.gov identifier: NCT02998957. Recruitment status: Recruiting. First posted: 21 December 2016. Last update posted: 2 October 2018. Trial registration dataset is available in Additional file 1. Protocol version 03. Issue date: 20 March 2018. Author: Carles Fernández.

## Background

Chronical obstructive pulmonary disease (COPD) is characterized by persistent and progressive airflow limitation associated with a chronic inflammatory response in the airways and lung parenchyma to noxious particles or gases [1]. COPD is a major cause of morbidity and mortality worldwide and its prevalence is expected to increase over the next decade because of continuous exposure to risk factors and the aging of the population [2]. It is estimated that in 2030 COPD will be the fourth leading cause of death globally [3]. Currently, the prevalence of the disease in Spain is 10.2% of the population between 40 and 80 years of age [4].

Dyspnoea, along with chronic cough and sputum production, is one of the main symptoms of COPD. Its severity and magnitude increase as the disease progresses and this disease is the main cause of disability and reduced quality of life in patients with COPD [5].

Patients may experience periods of acute exacerbation of COPD (AECOPD), defined as “a sustained worsening of the patient’s condition, from the stable state and beyond normal day-to-day variations, which is acute in onset and necessitates a change in regular medication in a patient with underlying COPD” [6]. These can be particularly severe exacerbations requiring hospitalization. In Spain, it is estimated that hospitalizations for AECOPD have an average cost of 344.96 euros per person per day [7], most of which is due to stays in hospital. These exacerbations are associated with 12% of mortality and 35% of readmissions up to 3 months after discharge [8].

Treatment of AECOPD includes bronchodilators, corticosteroids, antibiotics, oxygen therapy and non-invasive mechanical ventilation [9]. In the case of severe dyspnoea, oral or parenteral administration of opioids is recommended, although these have side effects and can cause respiratory depression [5]. However, dyspnoea remains a major symptom in patients hospitalized for an AECOPD [7].

The acupoint transcutaneous electrical nerve stimulation (AcuTENS) is a modern technique based on traditional acupuncture. It involves the stimulation of acupuncture points by using transcutaneous electrical nerve stimulation (TENS) instead of needles, which makes it a very-easy-to-learn, non-invasive method without the traditional acupuncture adverse effects such as infections or puncture of internal organs [10].

Several clinical studies using AcuTENS in patients with COPD have recently been published. All studies have used the stimulation of the acupuncture point named Dingchuan (EX-B1), traditionally used to treat dyspnoea. In those studies, a decrease in dyspnoea and an increase in forced expiratory volume in 1 second (FEV 1), β-endorphin level and quality of life have been observed [11–13]. However, these studies have been conducted on patients in stable condition, and little is known about the possible effect of this technique in patients with an AECOPD. Only one case study suggesting possible benefits for shortness of breath besides an increase in the level of oxygen saturation and β-endorphins has been published to date [14], and properly designed randomized placebo-controlled trials are needed to confirm these results.

The objectives of this study are to determine whether the use of AcuTENS at the Dingchuan point (EX-B1) could be beneficial for patients hospitalized for AECOPD by reducing dyspnoea, days of hospitalization, and consumption of regular medication. Moreover, we will assess possible adverse effects of this technique and its effect on mortality and readmissions at 3 months after discharge.

## Trial design

This is a multicentre, randomized controlled trial with blinded patients and assessors with a 1:1 allocation and two parallel groups. Trial registration dataset and SPIRIT 2013 checklist are available in Additional files 1 and 2.

## Methods

### Study setting

The study will be performed at the “Hospital del Mar” in Barcelona (Spain) and the “Hospital de Sant Joan de Déu de Manresa” in Manresa (Spain).

### Participants

The study subjects will be patients admitted at the study centres for an AECOPD. Participants will be recruited at the time of their admission by the pulmonology service and hospital emergency staff by using the following inclusion criteria: (1) patients between 45 and 75 years of age with a diagnosis of COPD in accordance with Global Initiative for Chronic Obstructive Lung Disease (GOLD) guidelines, (2) patients with one episode of hospitalization for COPD exacerbation in the past year but not more than three episodes, (3) smoking habit history of more than 10 packs a year, (4) patients able to correctly understand and answer the modified Borg scale, (5) patients with an initial degree of dyspnoea with a score of at least 5 in the modified Borg scale at recruitment, (6) patients recruited for the study during the first 48 h of hospitalization, and (7) patients who agree to participate in the study and sign the informed consent form.

Exclusion criteria will be (1) patients with any contraindication for transcutaneous electrical stimulation (patients with pacemakers, skin injury in the application area, etc.) and (2) patients with any cardiovascular, neurological or psychiatric disease that may affect the perception of dyspnoea.

### Interventions

**Fig. 1:**
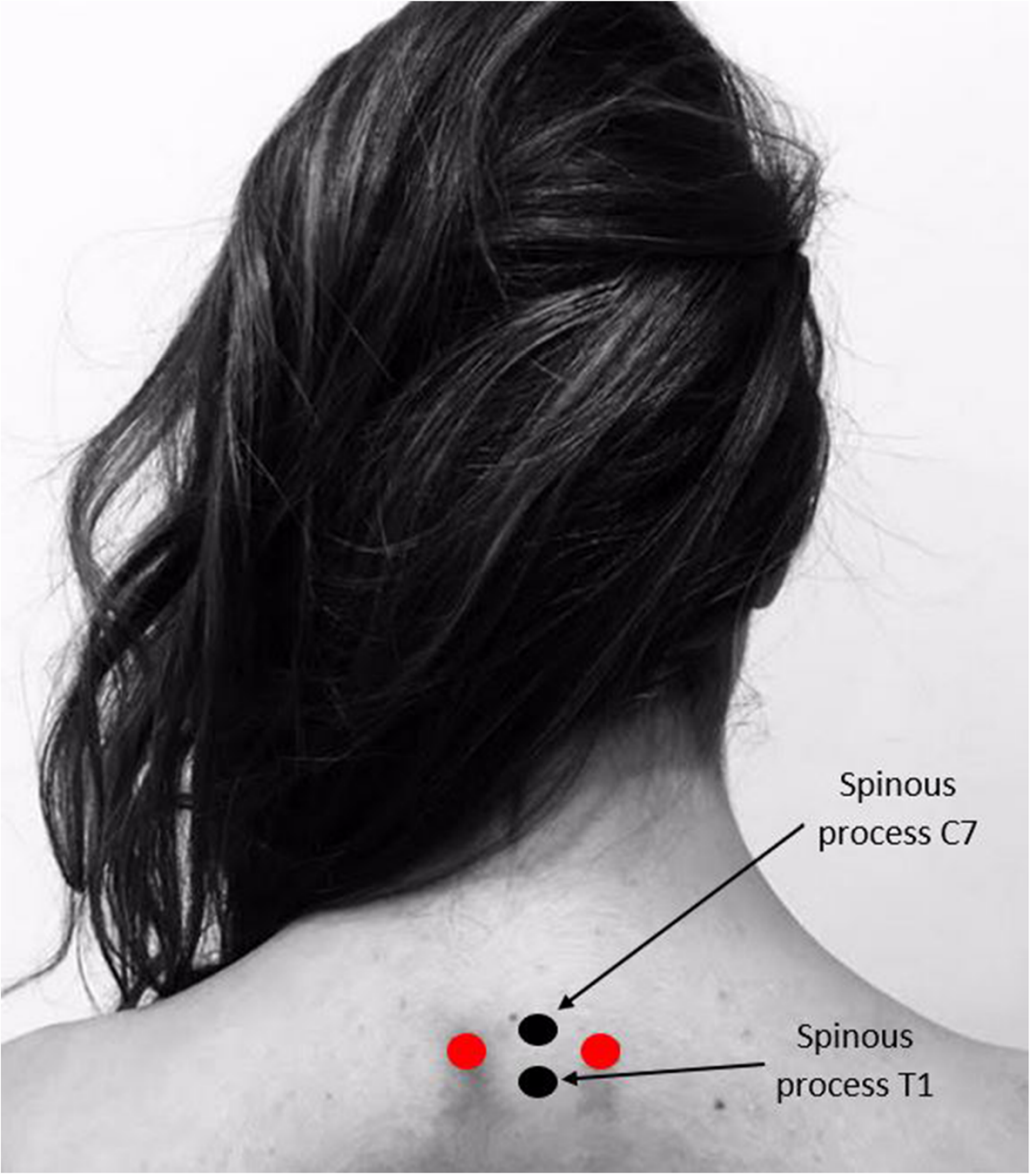
Location of Dingchuan acupuncture point

In both groups, the Dingchuan point (EX-B1) will be localized bilaterally at 0.5 “cun” from the midline between the posterior spinous processes of C7 and T1 (Fig. 1). Afterwards, a conductive plastic sheet between the skin and the electrodes will be placed. These sheets have a hole of about 0.5 cm in diameter, which will coincide with the point above. Finally, the electrodes will be fixed with tape.

In the AcuTENS group (experimental), the stimulation is performed by using a portable TENS electrostimulation device (Sale & Service TN23) that uses a biphasic rectangular wave with a frequency of 2 Hz and a pulse width of 200 mS. The stimulation will be achieved by using the highest intensity tolerated by the patient without pain.

The TENS simulated group (control) will have electrostimulators that are the same make and model as the experimental group but that have been modified to have no electrical outlet, even though the screen will light up and display the same data as in the unmodified device. Patients in this group will be informed that, owing to the frequency of stimulation, it is unlikely they will feel the electric stimulation.

In both groups, the session will last 45 min and will take place during a maximum of five consecutive days or until the patient is discharged.

If the acuTENS stimulation produces any kind of discomfort, the intensity of the current being applied to the patient will be reduced. If for any reason a treatment cannot be administered or is administered differently from the original protocol (e.g., only 30 min of treatment because the electrostimulation device had no battery), this will be reported. Any discomfort or skin reaction will be reported as an adverse event.

The interventions will be performed by physiotherapists or physicians who have a minimum of one year of clinical experience and who have been trained in the handling of the two treatment protocols. There will be a pre-pilot test to detect potential problems and ensure the adequacy of the implementation of the protocols.

Adherence to the treatment will be reported. No special stages for improving adherence will be taken since all patients will be hospitalized in the participating centres.

Both groups will receive the standard treatment for patients with AECOPD (bronchodilators, steroids, etc.) in accordance with medical criteria as well as the treatment with acuTENS or simulated acuTENS. No treatment is prohibited.

### Outcomes

#### Primary outcome measure

The primary outcome of this trial will be dyspnoea using the modified Borg scale. Evaluators will show a printed Borg scale to patients and explain the meaning of each punctuation and then patients will be asked to rate their dyspnoea. Data will be assessed at baseline and after each treatment session (days 1 to 5). Differences will be assessed by comparing groups’ mean scores at each day (days 1 to 5). The modified Borg scale is a valid and reliable assessment tool for dyspnoea in patients with an AECOPD [15].

#### Secondary outcome measures

Hospitalization length will be measured by using the number of days from the time of admission until the patient’s discharge. Scores will be compared by using mean differences between groups.

Peak expiratory flow will be assessed by using a peak flow meter and using litre per minute units at baseline and after each treatment session. For each assessment, peak flow will be measured three times and only the highest score will be considered. Differences will be assessed between groups by using mean scores at each day (days 1 to 5).

Adverse events will be recorded after each treatment session; the evaluator will ask the patient for any adverse event related to the intervention. Description of each event and its frequency will be described for each group.

Blood gas analysis—partial pressure of oxygen (PaO_2_), partial pressure of carbon dioxide (PaCO_2_), arterial blood pH, bicarbonate and oxygen saturation (SaO_2_)—at days 1 to 5 will be recorded if available in the patient’s clinical history since no specific analysis will be made for the trial. Differences will be assessed by comparing means at each day (days 1 to 5).

Relapse and readmissions at 3 months after discharge will be assessed by using the participant’s clinical history. Mean number of relapses and readmissions during the 3-month period after discharge will be compared between groups.

Mortality will be assessed by using the ratio of patients who died during the 3-month period after discharge in each group.

Quantity of drugs administered during patient’s hospitalization will be extracted from the patient’s clinical history. Mean dosages will be compared between groups. Data will be collected by using a standard form designed by the study investigators, and database entry will be double-checked.

### Participant timeline

**Fig. 2:**
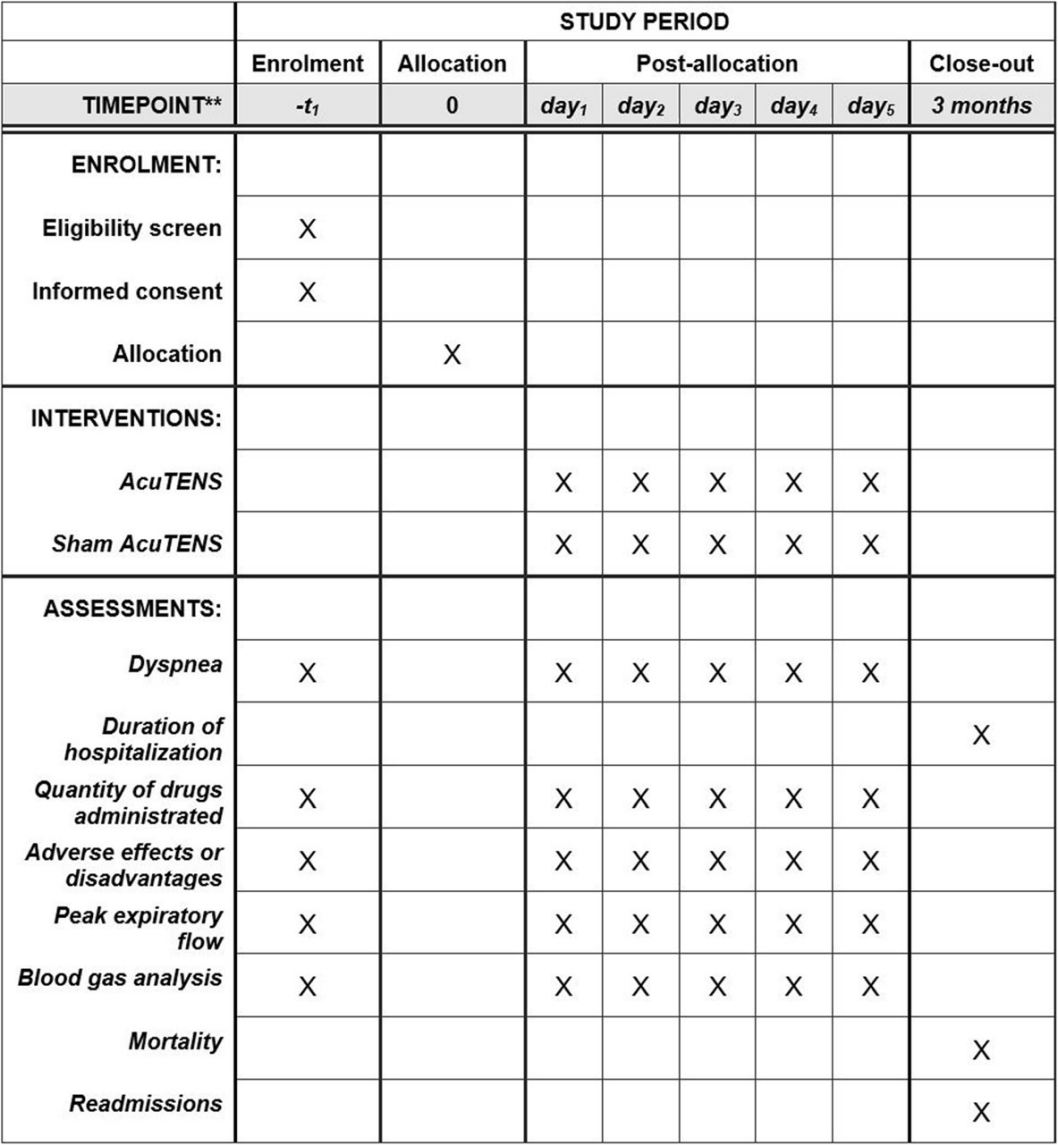
Schedule of enrolment, interventions and assessments

Participant timeline is described in Fig. 2.

### Randomization and allocation

Participants will be randomly assigned to the acuTENS or sham acuTENS group with a 1:1 allocation by using a computer-generated randomization list. A blocked randomization list will be generated for each centre by the main author, who will not be involved in recruitment or assessment. Randomization lists will be kept by using an electronic key and will be available only to the persons responsible for administering the treatments.

Once participants give consent for participation, a consecutive number will be assigned by the head of recruitment, and the staff responsible for administrating the treatment will match the patient’s number with the randomization list to define the patient’s allocation.

### Blinding

Trial participants, outcome assessors and usual care providers will be blinded during the trial, and only the physiotherapists who will perform the acuTENS or sham acuTENS procedure will know the patient’s allocation. To ensure that they remain blinded to allocation, the medical staff will not be present during the application of technology. There will be no circumstances under which unblinding is permissible. Data analysis will be performed by a blinded external statistician.

### Sample size

Considering a minimal clinically important difference regarding dyspnoea, measured by the Borg scale, of 2 [16] and assuming a common standard deviation of 2.6, an α error of 0.05, a β error of 0.2 and a 10% rate of losses, we calculate that we will need to recruit 60 patients for this study. To reach this target sample size, pulmonology service and hospital emergency staff will be informed so they can detect possible candidates.

### Statistical methods

There will be a descriptive analysis of the demographic data by using means and standard deviations (mean ± standard deviation) for continuous variables and absolute values and percentages for qualitative outcomes.

For the comparative analysis between the two groups, continuous outcomes (such as Borg scale score and number of hospitalization days) will be analysed by using mean differences with a 95% confidence interval with a Student *t* test or Mann–Whitney *U* test depending on the distribution of these. For dichotomous outcomes (such as mortality at 3 months), risk ratio will also be used with a confidence interval of 95% and chi-squared test.

The main analysis will be carried out by intention to treat, but there will be another per-protocol analysis for patients who have received at least three interventions. A *P* value of less than 0.05 will be considered significantly different.

In case of missing data, last value carried forward methods will be used. In case of significant baseline differences between the two groups, there will be an adjusted analysis for these outcomes.

## Discussion

This protocol describes the design of a clinical trial investigating the effectiveness of adding acuTENS stimulation to usual treatment of dyspnoea in patients hospitalized with an AECOPD. If the results of this trial are positive, a new non-pharmacological and inexpensive intervention could be used in cases of intense dyspnoea. Moreover, dyspnoea improvement could lead to a reduction in medication and hospitalization days, reducing hospitalization costs. It is also important to highlight that this is quite a simple intervention to learn and TENS devices are usually available in any health centre; this is important as it would facilitate the technique’s implementation and generalization.

## Trial status

Protocol Admen Number: 03. Issue date: 20 March 2018. Recruitment start day: 1 April 2018. Estimated recruitment completion date: June 2020.

## Supplementary information

**Additional file 1.** Trial registration dataset.

**Additional file 2.** Standard Protocol Items: Recommendations for Interventional Trials (SPIRIT) 2013 Checklist: Recommended items to address in a clinical trial protocol and related documents*.

## Data Availability

The datasets used or analysed (or both) during this study are available from the corresponding author on reasonable request.

## Abbreviations

AcuTENS: Acupoint transcutaneous electrical nerve stimulation
AECOPD: Acute exacerbation of chronic obstructive pulmonary disease
COPD: Chronical obstructive pulmonary disease
TENS: Transcutaneous electrical nerve stimulation
