# Supplementary material for: Acupoint transcutaneous electrical nerve stimulation in hospitalized COPD patients with severe dyspnoea: study protocol for a randomized controlled trial
Source: Trials. 2019 Dec 11;20:707. doi: 10.1186/s13063-019-3757-x (PMC6907236; doi:10.1186/s13063-019-3757-x)
Supplement: Supplementary file 1 — Additional file 1. Trial registration dataset. [file 13063_2019_3757_MOESM1_ESM.docx]

| **Data category** | **Information** |
| --- | --- |
| Primary registry and trial identifying number | ClinicalTrials.gov NCT02998957 |
| Date of registration in primary registry | November 30, 2016 |
| Secondary identifying numbers | PIC-195-15 |
| Source(s) of monetary or material support | Professional College of Physiotherapists of Catalonia and the Scientific Society of Acupuncture of Catalonia and the Balearic Islands |
| Primary sponsor | Fundació Sant Joan de Déu |
| Secondary sponsor(s) | - |
| Contact for public queries | Carles Fernández [carlesfj@blanquerna.url.edu] |
| Contact for scientific queries | Carles Fernández [carlesfj@blanquerna.url.edu] School of Health Science Blanquerna, Ramón Llull University, Barcelona, Spain. |
| Public title | Acupoint Transcutaneous Electrical Nerve Stimulation in Hospitalized COPD Patients with Severe Dyspnoea |
| Scientific title | Efficacy of Transcutaneous Electrical Stimulation at Dingchuan (EX-B1) in Hospitalized COPD Patients with Severe Dyspnoea: Patient and Assessor Blinded Randomized Placebo Control Trial |
| Countries of recruitment | Spain |
| Health condition(s) or problem(s) studied | Chronic Obstructive Pulmonary Disease (COPD) |
| Intervention(s) | Stimulation of acupuncture point Dingchuan using transcutaneous electrical nerve stimulation (TENS), 1 session a day for 5 consecutive days. |
|  | Placebo comparator: Portable TENS electrostimulation device with no electrical output |
| Key inclusion and exclusion criteria | Patients aged between 45 and 70 years, with a diagnose of COPD according to the GOLD guidelines  Patients with one episode of hospitalization for COPD exacerbation in the past year, but not more than three episodes |
|  | Smoking habit history of more than 10 packages-year.  Patients able to correctly understand and answer the modified Borg scale  Patients with an initial degree of dyspnoea with a score of at least 5 in the modified Borg scale  Patients recruited for the study during the first 48 hours of their hospitalization  Patients who accept to participate in the study and sign the informed consent  Exclusion Criteria:  Patients with any contraindication for transcutaneous electrical stimulation (patients with pacemakers, skin injury in the application area ...). |
|  | Patients with any cardiovascular, neurological or psychiatric disease that may affect the perception of dyspnoea. |
| Study type | Interventional |
|  | Allocation: randomized intervention model. Parallel assignment masking: double blind (subject, outcomes assessor and medical staff) |
|  | Primary purpose: treatment |
|  |  |
| Date of first enrolment | April 2018 |
| Target sample size | 60 |
| Recruitment status | Recruiting |
| Primary outcome(s) | Dyspnoea at days 1 to 5 using the modified Borg scale |
| Key secondary outcomes | Length of hospitalization, peak expiratory flow (day 1- day5), relapses, readmissions and mortality at 3 months after discharge, adverse events, blood gas analysis Blood gas analysis (PaO2, PaCO2, arterial blood pH, bicarbonate and SaO2) at days 1 to 5, Quantity of drugs administer during patient’s hospitalization |
